# Effect of Melittin Complexes with Graphene and Graphene Oxide on Triple-Negative Breast Cancer Tumors Grown on Chicken Embryo Chorioallantoic Membrane

**DOI:** 10.3390/ijms24098388

**Published:** 2023-05-07

**Authors:** Karolina Daniluk, Agata Lange, Barbara Wójcik, Katarzyna Zawadzka, Jaśmina Bałaban, Marta Kutwin, Sławomir Jaworski

**Affiliations:** Department of Nanobiotechnology, Institute of Biology, Warsaw University of Life Sciences, 02-786 Warsaw, Poland; karolina_daniluk@sggw.edu.pl (K.D.); agata_lange@sggw.edu.pl (A.L.); barbara_wojcik@sggw.edu.pl (B.W.); jasmina_balaban@sggw.edu.pl (J.B.); slawomir_jaworski@sggw.edu.pl (S.J.)

**Keywords:** melittin, nanomaterials, breast cancer, drug-delivery

## Abstract

One of the components of bee venom is melittin (M), which has strong lysing properties on membranes. M has high toxicity to cancer cells, but it also affects healthy cells, making it necessary to use methods for targeted delivery to ensure treatment. This research is a continuation of previous studies using graphene nanomaterials as M carriers to breast cancer cells. The studies described below are conducted on a more organized biological structure than what is found in vitro cells, namely, cancerous tumors grown on a chicken embryo chorioallantoic membrane. Caspase 3 and 8 levels are analyzed, and the level of oxidative stress markers and changes in protein expression for cytokines are examined. The results show that M complexes with nanomaterials reduce the level of oxidative stress more than M alone does, but the use of graphene (GN) as a carrier increases the level of DNA damage to a greater extent than the increase caused by M alone. An analysis of cytokine levels shows that the use of the M and GN complex increases the level of proteins responsible for inhibiting tumor progression to a greater extent than the increase occasioned by a complex with graphene oxide (GO). The results suggest that the use of GN as an M carrier may increase the toxic effect of M on structures located inside a cell.

## 1. Introduction

It is estimated that 1 in 8 women in the U.S., or approximately 13% of American women, will develop malignant breast cancer in their lifetime. Currently, malignant breast cancer is one of the main health problems affecting women due to its high rate of mortality and morbidity. Moreover, the disease is related to globalization because its incidence is significantly greater in high-income countries compared to low-income countries [1,2].

Due to the great diversity of biological subtypes that show differences at the molecular and clinical level, the name “breast cancer” is used to identify a large group of cancer types [3,4]. This group includes triple-negative breast cancer, which does not express human epidermal growth factor 2 receptor, estrogen, and progesterone receptors and that accounts for approximately 15% of invasive breast cancer [5,6]. 

There are fewer treatment options for triple-negative breast cancer than for other breast cancer subtypes due to the lack of the above-mentioned receptors, which are often therapeutic targets. The most common method of treatment for this type of cancer is chemotherapy, which destroys the patient’s entire body; therefore, new methods of delivering drugs and therapeutic agents are constantly being sought [7,8].

Honey bee venom has been used since ancient times to treat various health ailments, including rheumatism and skin diseases. It is composed of various ingredients that give it antiviral, antibacterial, anti-inflammatory, and antimutagenic effects [9,10,11]. The most important components of the venom include apamin, the enzyme phospholipase A2, and the proteases API SI and API SII. 

However, the main component of bee venom is melittin (M), which makes up 50–75% of its dry weight [12,13]. M is a peptide made of 26 amino acids, the arrangement of which gives it an amphiphilic character, which means that it is both hydrophobic and hydrophilic. The amino terminus imparts hydrophobic properties, and the carboxyl terminus has a positive charge [14]. The biological properties of M are based on the mechanism of its interaction with biological membranes. M binds to the negatively charged lipid bilayer in a biological membrane and disrupts its continuity by creating pores made of M tetramers. The effect of M action is the disintegration of biological membranes; in the case of a cell structure, this often leads to cell death [15,16].

Due to differences in the potential of the membranes of healthy and cancer cells, the use of M as an anticancer agent has been investigated. M’s anti-cancer effect has been demonstrated in many types of cancer, such as prostate, lung, and ovarian cancers [17,18,19].

In the case of breast cancer, M also exhibits dose-dependent toxicity, induces cell death by apoptosis and necrosis, and inhibits cell emigration [20,21,22]. While the lytic action of M is recognized as being non-selective and acting on any type of biological membrane, this peptide has been shown to be selective for triple-negative breast cancer and to induce cell death with a low effect on normal cells [23]. However, to exploit the anticancer potential of M while maintaining safety for healthy cells, a drug delivery system must be used.

Despite the promising anticancer properties of M, further research is needed to fully understand its potential in cancer treatment. In particular, additional studies are needed to evaluate the safety and efficacy of M in human trials. The anti-cancer properties of M make it an interesting area of research for the development of new cancer therapies. In recent years, there has been increasing interest in the use of nanotechnology for drug delivery, including the delivery of M to cancer cells. Nanoparticles can enhance the delivery and effectiveness of therapeutic agents by improving their solubility, stability, and bioavailability [24,25]. Moreover, nanoparticles can be designed to selectively accumulate in tumors, thereby increasing the therapeutic index of a drug [26].

The use of nanoparticles as M carriers for cancer cells can reduce M off-target effects. Several studies have reported on the use of nanotechnology for the treatment of cancer, including breast cancer. A study using gold nanoparticles as M-loaded niosomes showed that complexes inhibited the growth of breast cancer cells in vitro to a significantly greater extent compared to free M [27]. Among nanomaterials, carbon nanoparticles show the potential to deliver M [28,29]. 

It has been shown that carbon-based nanomaterials are taken up into cancer cells by internalization through various cellular transport mechanisms, including endocytosis [30]. Thus, potentially, M in complex with GN or GO can be taken up by cells via the aforementioned transport mode and act inside the cell. Additionally, GN and GO nanomaterials seem to be safe and effective drug carriers due to their size, biological properties, and easy surface functionalization [31]. Studies show that GN nanomaterials increase the permeability of cancer cell membranes, which may contribute to increased absorption of therapeutic compounds [32,33].

Studies were carried out in which the anticancer potential of GO in the hemin and chlorin e6 nanoplatform against breast cancer was checked. It was shown that the highest concentration of the studied complex with GO was found in the tumor. Lower fluorescence intensity of the complex was observed in the kidneys, liver, and lungs of the tested mice, and a negligible signal was observed in the spleen and heart. In addition, the histopathological analysis did not show any significant abnormalities, which allows us to make conclusions about its biological safety [34]. On the other hand, GO itself inhibits tumor growth and reduces migration of breast cancer cells. In addition, it does not show unequivocal toxicity to healthy cells [35,36].

Studies using GN and GO show that their cytotoxic effect is dose-dependent and increases with the concentration of the nanomaterial, but the degree of toxicity also depends on the type of cells [37,38,39,40,41].

Our previous studies examined the effect of M complexes with GN and GO compared to the effect of M alone on breast cancer cell lines. The use of the complexes was shown to significantly reduce the level of necrosis compared to the level occasioned by M alone, which may be related to the reduction of inflammation in a tumor environment in response to the action of a potential drug [22]. Our next study showed that, in MDA-MD-231 cells, complex MGN is transported to cells via caveolin-dependent endocytosis, and MGO complex can be transported to breast cancer cells in various ways. The use of GN nanoparticles as carriers did not reduce toxicity to healthy cells; however, these investigations were in vitro studies with a monoculture. In addition, the results of previous studies indicate the potential use of GN and GO as carriers due to their electrostatic interaction with M and the high degree of M entrapment efficiency [42]. In this research, the response of tumors cultured on chicken embryo chorioallantoic membrane (CAM) to treatment with the tested complexes and to M alone was examined. In the described studies, the cells did not grow in a monoculture but were in contact with other cells and factors, for example, in pro-angiogenic tumor vessels, which could have affected the results of studies using the same concentrations of the compounds tested in vitro. We examined the impact of MGN and MGO complex by analyzing the mass of tumors, the activity of caspase 3 (CASP3) and caspase 8 (CASP8), the expression of pro-inflammatory proteins at the protein level, and the levels of malondialdehyde (MDA), superoxide dismutase (SOD), and glutathione (GHS).

## 2. Results

### 2.1. Characterization of Complexes

**Figure 1:**
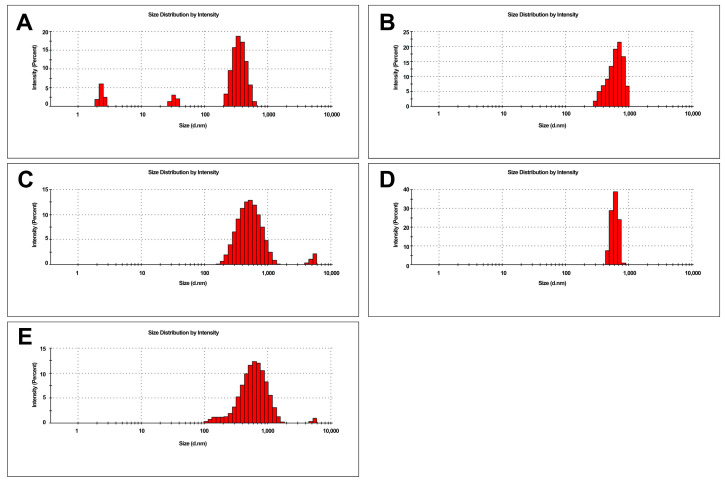
Size distribution of M, GN, GO, and complexes. (**A**)—M; (**B**)—GN; (**C**)—GO; (**D**)—MGN; (**E**)—MGO.

**Figure 2:**
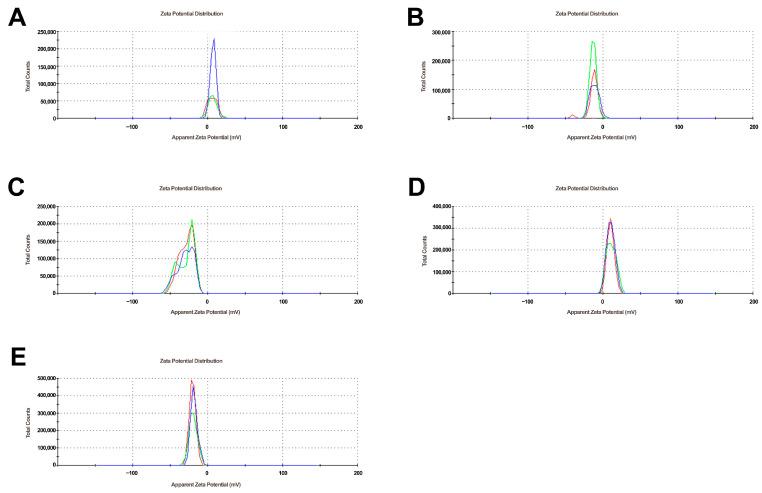
Zeta potential of M, GN, GO, and complexes. (**A**)—M; (**B**)—GN; (**C**)—GO; (**D**)—MGN; (**E**)—MGO. Three different colors mean repetitions.

DLS analyses showed the presence of GN structures in a range of three fractions, as GO observed one fraction reaching the size of 1000 nm (Figure 1). Zeta potential analysis showed a positive charge for the M alone and a negative charge for the nanoparticles alone (Figure 2), which confirms their physical bonding in complexes [43].

### 2.2. Tumor Mass and Volume

No statistically significant differences in the mass and volume of tumors were found. Breast cancer tumors, in contrast to glioblastoma tumors, do not form compact tumors in vivo. Therefore, these parameters may not be correlated with the effect of the tested substances after 3 days of exposure.

### 2.3. Activity of Caspases

**Figure 3:**
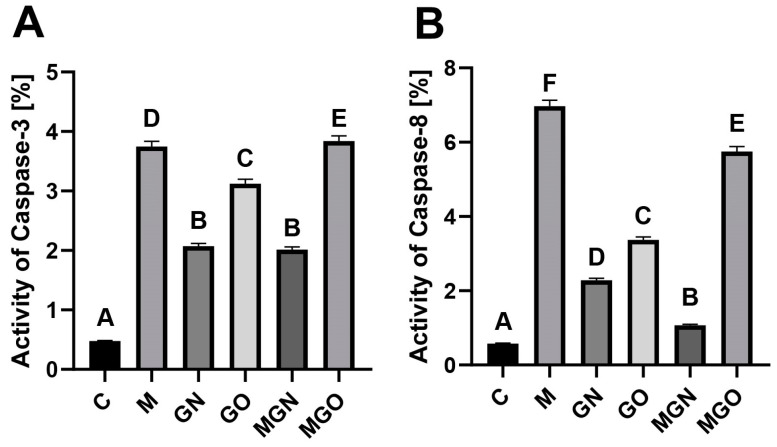
Activity of caspase 3 (**A**) and caspase 8 (**B**) in breast cancer tumors. A–F results in one group with different indexes are significantly different (*p*-value < 0.05).

The results of ELISA tests of CASP3 and CASP8 activity are presented in Figure 3. A significant increase in the activity of both caspases was observed in all tested groups compared to the control. For CASP3 and CASP8, the greatest increase occurred after treatment with M and MGO breast cancer tumors.

### 2.4. Analyses of Oxidative Stress Markers

**Figure 4:**
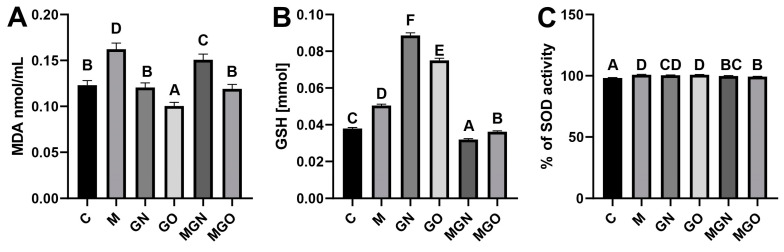
Analysis of oxidative stress markers in breast cancer tumor lysate. (**A**) Malonaldehyde concentration; (**B**) Superoxide dismutase activity; (**C**) Glutathione level; A–F results in one group with different indexes are significantly different (*p*-value < 0.05).

Three markers of oxidative stress were tested, and the results are shown in Figure 4. There was a significant increase in the concentration of MDA in the group treated with M and MGN, and a significant decrease in the group treated with GO. In the analysis of SOD activity, a significant increase was observed for all groups compared to the control. There was a significant increase in GSH in the groups treated with M, GN, and GO, and a significant decrease in the group exposed to MGN and MGO.

### 2.5. Measurement of 8-OHdG Concentrations

**Figure 5:**
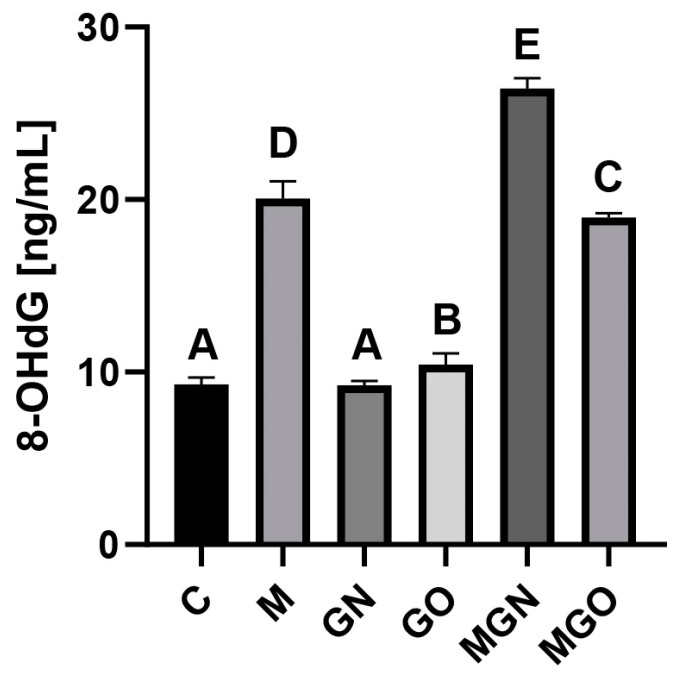
8-OHdG concentration in breast cancer tumor lysate. A–E results in one group with different indexes are significantly different (*p*-value < 0.05).

A concentration analysis showed a significant increase in the 8-OHdG marker in the M, GO, MGN and MGO-treated groups (Figure 5). Exposure of breast cancer tumors to GN did not change the concentration of this marker.

### 2.6. Cytokine Protein Level

**Figure 6:**
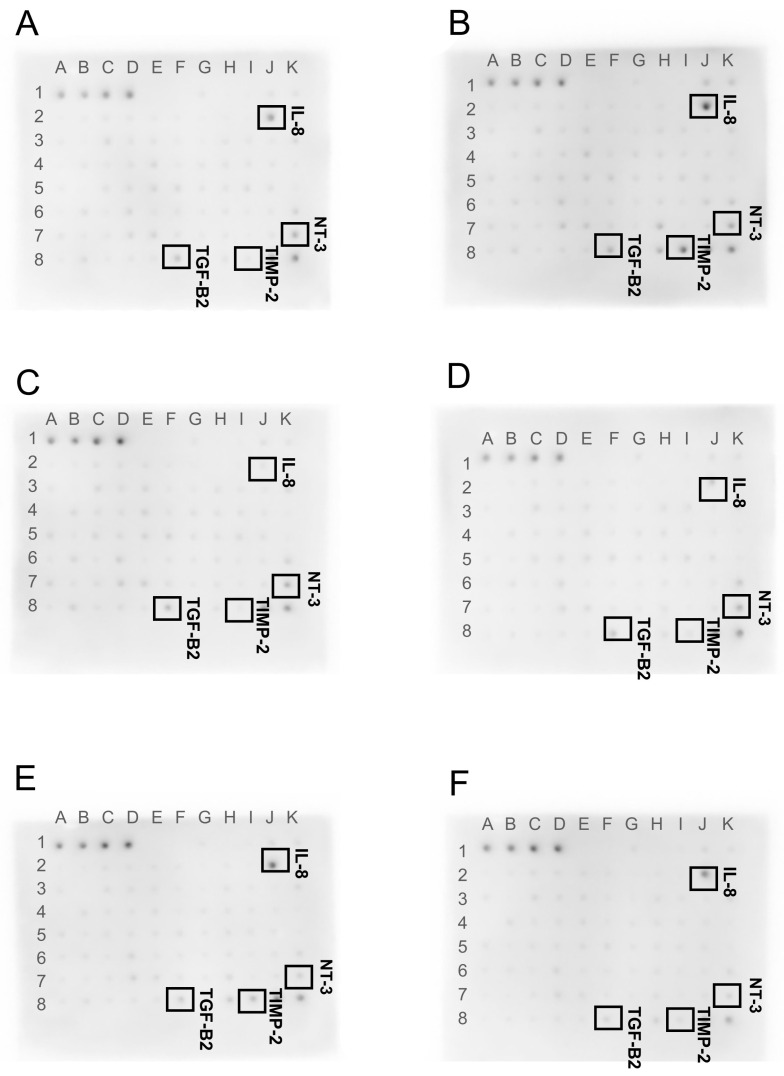
Antibody array analysis of the Human Cytokine Antibody Array (original drafts) in MDA-MB-231 cells. (**A**) control group, (**B**) M-treated group, (**C**) GN-treated group, (**D**) GO-treated group, (**E**) MGN-treated group, and (**F**) MGO-treated group.

Results from the Human Cytokine Antibody Array are shown in Figure 6. Changes in the protein expression of four cytokines: ilterleukin-8 (IL-8), neurothropin-3 (NT-3), tissue inhibitor of metalloproteinases 2 (TIMP-2), and tumor necrosis factor beta-2 T (NF-B2) were observed. Results were normalized and compared to a dots control sample.

## 3. Discussion

In our study, we used carbon nanoparticles as a potential M carrier to reach breast cancer cells. Previous studies reported the characteristics of the formed complexes and their components in terms of physicochemical aspects and showed that M combines with GN and GO via electrostatic bonds to form complexes. The level of encapsulation of M with GN was 86% and with GO it was 78%, which is a suitable indicator for potential therapeutic agents. It has been proven that MGN is transported to MDA-MB-231 cells through caveolin-dependent endocytosis, while MGO is transported in multiple ways [42]. In addition, the use of carbon nanoparticles reduced the rate of cell death by necrosis to a greater extent compared to the reduction resulting from M alone [22]. In this study, GN and GO were investigated as carriers of M to a tumor, which has a more organized structure than cell culture and which was cultured from the MDA-MB-231 triple-negative breast cancer cell line by analyzing CASP3 and CASP8 activity, oxidative stress, and expressing cytokine proteins.

Previous in vitro studies showed that there was an increase in CASP3 and CASP8 at the protein level in the MDA-MB-231 cell line after exposure to the MGN complex. On the other hand, studies of the activity of the same caspases on tumors showed an increase in CASP3 activity relative to the control in all the studied groups, while a minimal increase in activity relative to the M-treated group was noted in the MGO group. Moreover, the results indicated a large increase in CASP3 activity both in response to M alone and to GO and MGO. In the case of MGN and GN treatment, the level was similar to that of M alone and to that of GO and MGO, but was lower than the level after treatment with M alone. CASP8 activity was high in the case of M alone and when treated with the MGN complex, but in both cases, it was lower than the level for M alone.

The obtained results indicate a different response to the tested complexes at the in vitro and in vivo levels. CASP3 is involved in the activation of apoptosis through both the mitochondrial and extrinsic pathways (Figure 3A). In contrast, CASP8 is involved in the extrinsic apoptosis pathway. Based on the results, it can be concluded that, in the case of organized biological structures such as CAM-grown tumors, MGO and M can cause tumor cell death via the extracellular apoptosis pathway (Figure 3B). However, in the case of MGN, cell death may be activated by the mitochondrial apoptotic pathway to a greater extent than for MGO, which may mean that M has a greater effect on intracellular structures than it does in a complex with GO. Other studies have linked GO as a vehicle that binds to a cell’s death receptor and activates the extracellular apoptosis pathway [44].

Oxidative stress has been associated with all stages of progression and development of most cancers as well as the effectiveness of applied therapies [45]. Therefore, we performed an analysis of three markers of oxidative stress, namely MDA, SOD, and GSH.

Oxidative stress results in damage to cells, tissues, and even entire organs. High levels of reactive oxygen species and free radicals, especially over the long term, can lead to lipid damage [46]. When lipids undergo peroxidation, additional toxic radicals are formed as a result of the oxidation of unsaturated fatty acids. Oxidizing agents attack the fats that make up cell membranes to form lipid peroxides. These compounds then react to form malonaldehyde (MDA), which is one of the most commonly studied compounds used to assess the impact of oxidative stress on lipids.

The formation of breast cancer is associated with oxidative stress. Studies show an increase in MDA in patients with breast cancer [47,48]. The results of this research showed a significant increase in the level of MDA in the group treated with M and MGN (Figure 4A). The results suggest that MGN can damage tumor cells through lipid damage and induce oxidative stress.

SOD is an antioxidant enzyme that plays a critical role in protecting cells from oxidative damage by catalyzing the conversion of superoxide radicals into hydrogen peroxide and oxygen [49,50]. An increase in SOD activity relative to the control was noted in all groups, while complexes caused a lower increase than their component nanomaterials alone (Figure 4B).

GSH is known as one of the most important factors involved in antioxidant processes [51]. Several cancers, including ovarian and lung cancer, have elevated levels of GSH [52]. Many publications report that increased levels of this factor in cells promote the initiation and progression of cancer [53,54]. On the other hand, increased levels of GSH may be a response to the toxic effects of the tested factor [55,56]. In the present study, GSH levels increased significantly in group M (by approximately 30%), GN (by approximately 130%), and GO (by approximately 100%), while a decrease relative to the control was seen in the MGN group (by approximately 15%) (Figure 2C).

The results obtained suggest that nanomaterials and M alone cause greater oxidative stress than what is caused by the tested complexes, which may be a crucial factor at the level of the body’s response to a potential therapeutic agent. These results are consistent with the results of SOD activity, where the nanomaterials alone caused a higher level of oxidative stress than what was caused by the complexes.

The 8-OHdG marker is a factor that allows the assessment of oxidative stress in cells and provides details about DNA damage caused by the tested factor. Studies have shown that M has a cytotoxic effect on breast cancer cells [57]. Researchers investigated whether the use of carriers made from GN nanomaterials would affect M’s toxic effect on DNA. The results indicated that M alone increased the level of 8-OHdG by approximately 2-fold (up to 20 ng/mL) compared to the control in breast cancer cells, while the MGN complex raised it to approximately 25 ng/mL. However, in the case of the MGO complex, the level of 8-OHdG was similar to the level after exposure to the M peptide alone (Figure 5). These results indicate that the MGN complex may have a toxic effect mainly on intracellular structures, which confirms the previously described findings. In addition, this marker is involved in the induction of apoptosis, so its increase in the level in the MGN group may be related to the activation of apoptosis in the intrinsic pathway, which is reflected in the results of CASP3 activity [58].

The membrane assay revealed differences in the expression of four cytokines (Figure 6). One of them, IL-8, is characterized by its increased expression in breast cancer cells compared to healthy tissue, especially in HER2-positive tumors [59]. Many studies have shown that the increase in IL-8 expression is associated with heightened breast cancer metastasis [60,61]. Il-8 is known as a chemotactic agent for granulocytes and neutrophils, and it is also believed to trigger a pro-inflammatory response in cancer [59].

Our study showed an increase in the expression of the IL-8 protein in tumors treated with M, MGN, and MGO and a decrease in the groups exposed to only carbon nanoparticles included in the study compared to the control group. IL-8 is responsible for the mobilization of the tumor microenvironment for the tumor’s survival, which may indicate the activation of defense mechanisms against the negative effect of the tested complexes on the tumor [62].

NT-3 is a protein belonging to the neurotrophin family. NT-3 functions as a growth factor that stimulates the development and differentiation of new neurons and synapses while promoting the survival and differentiation of existing neurons in both the peripheral and central nervous systems [63]. It has been shown that NT-3 has another function, which is promoting the metastatic growth of breast cancer cells in the brain. Firstly, NT-3 promotes the mesenchymal–epithelial transition (MET) of breast cancer cells to an increasingly epithelial-like phenotype and dramatically enhances the ability of these reverted breast cancer cells to proliferate in the brain. Consequently, knocking down NT-3 in epithelial-like breast cancer cells leads to a partial reversion to an epithelial–mesenchymal transition (EMT)-like phenotype and a dramatic reduction of metastatic tumor burden in the brain. NT-3-induced mesenchymal–epithelial reversion also coincides with reduced migratory ability, increased expression of human epidermal growth factor receptor 2 (HER2) and E-cadherin at the cell–cell junction, and reduced expression of EMT-inducing transcription factors, such as Snail, in the MET-reverted breast cancer cells [64,65].

In the studies conducted, a decrease in expression at the protein level for NT-3 was observed in the groups treated with MGN and MGO complexes. Observed changes may indicate a decrease in the ability of tumors created from the MDA-MB-231 cell line to metastasize, especially to the brain. A triple-negative breast cancer cell line was used in the study, and this type of cancer is likely to metastasize [66]. Thus, the use of the tested M complexes may reduce or eliminate breast cancer metastasis.

TIMP-2 belongs to the family of tissue metalloproteinase inhibitors (TIMPs), which are involved, among others, in inhibiting cell proliferation and migration by blocking matrix metalloproteinases (MMPs) [67]. This study indicates that cancer cell migration may be induced by an imbalance between MMP and TIMP. However, TIMP-2 has a different mechanism of action from MMP; some of its functions have been shown to function independently from MMPs, and it can inhibit tumor growth and angiogenesis and may reduce the migration and invasiveness of cancer cells [68,69]. At the same time, TIMP-2 is responsible for blocking some MMPs, such as MMP-2, which contribute to metastasis. The use of TIMP-2 in the treatment of triple-negative breast cancer has been shown to suppress the cellular pathways involved in tumor metastasis [70].

In this study, an increase in expression at the level of this protein was noted in the group treated with M and MGN. Breast cancers are characterized by low expression of TIMP-2, and elevated levels of TIMP-2 are associated with a poor prognosis [71]. However, an increase in protein expression in response to M and MGN treatment may be linked to a reduction in tumor growth and an attenuation of tumor vascularization, for the MGN treatment group, and a decreased metastatic capacity in the body.

TGF-B2 has been shown to play a complex role in the development and progression of breast cancer. Although it acts as a tumor suppressor in healthy tissues, it can promote tumor growth and metastasis in breast cancer [72]. Several studies have investigated the role of TGF-B2 in breast cancer and found that it promotes breast cancer cell invasion and metastasis by inducing the expression of MMPs that degrade the extracellular matrix and allow cancer cells to migrate [73]. TGF-B2 has also been shown to play a role in the development of breast cancer stem cells, which are able to self-renew and differentiate into all the different types of cells that make up the tumor. Research shows that TGF-B2 promotes the survival of breast cancer stem cells by upregulating the expression of the anti-apoptotic protein Bcl-2 [74]. A study found that TGF-beta2 protects breast cancer stem cells from the effects of chemotherapy by upregulating the expression of multidrug resistance proteins [75]. TGF-B2 has also been shown to modulate the immune response in breast cancer: TGF-B2 suppresses the activity of cytotoxic T cells, which are responsible for recognizing and destroying cancer cells [76].

This study showed that TGF-B2 protein expression decreased in both M complexes. Based on the above study, it can be inferred that the use of these complexes may reduce the ability of a tumor to metastasize and weaken the defense mechanisms of cancer stem cells.

To summarize, the results obtained show that the tested complexes induce different responses in a breast cancer tumor grown from MDA-MB-231 cells. Upon analyzing the data, it can be inferred that GN is a more promising carrier of M than GO. MGN did not activate the extrinsic apoptosis pathway to the same extent as M and MGO, so perhaps M associated with GN had a toxic effect mainly on extracellular structures. MGN also reduces inflammation compared to M alone. In addition, MGN complex increased the expression of more factors that inhibit metastasis and tumor growth than MGO. It is worth emphasizing that the effects of the studied complexes are the result of simultaneous exposure to the nanomaterial and M and their interaction as factors harmful to cancer cells. The use of GN and GO allows the liberation of M by the nanomaterial, which has been confirmed in previous studies [42], and the reduction of its spread outside the tumor when administered into the tumor. Oxidative stress can cause the production of cytokines, which in turn are used to communicate with other cells [77]. Most cancers are characterized by increased rates of oxidative stress compared to healthy cells, which is conducive to, among others, mutations [76,78]. However, the induction of oxidative stress in cancer cells above their programmed level causes oxidative damage to DNA and lipids and initiates cell death by apoptosis. As a defensive response, the cells activate defense systems that resist the production of antioxidants, including SOD and GSH. Currently, the mechanism based on inducing an increase in oxidative stress as an anticancer effect is used in many therapies [79,80,81,82,83,84,85,86]. The obtained results suggest that, in the case of a potential therapy that induces oxidative stress in breast cancer tumors, the MGN complex seems to be the most promising. In conclusion, the combination of M and nanomaterials holds great promise for the treatment of cancer, including breast cancer. Further research is needed to optimize the design and formulation of nanoparticle–M conjugates, as well as to evaluate their safety and efficacy in clinical trials.

## 4. Materials and Methods

### 4.1. Preparation of Complexes and Characterization

Pure M peptide was obtained from Sigma-Aldrich (purity ≥ 85%; Munich, Germany) in powder form and dissolved in ultrapure water. GN (thickness: 6–8 nm; purity > 99.5%) powder was purchased from Skyspring Nanomaterials (Houston, TX, USA), and GO (thickness < 2 nm; purity 98%; diameter: 8–15 µm) powder was purchased from the Institute of Electronic Materials Technology in Warsaw, Poland. M was added to each type of nanomaterial to obtain 2 different complexes in concentrations of 20 μg/mL for GN and GO and 10 μg/mL for M.

To obtain complexes by self-organization, the complexes were incubated at room temperature and vortexed for 15 min. Size distribution (dynamic light scattering method) and Zeta potential (laser Doppler electrophoresis method) of M GN, GO, and colloid complexes were measured using a Zetasizer Nano ZS, model ZEN3600 (Malvern Instruments, Malvern, UK).

### 4.2. Cell Culture and Culture of Tumors on a Chorioallantoic Membrane

The human breast adenocarcinoma MDA-MB-231 cell line was obtained from American Type Culture Collection (Manassas, VA, USA) and maintained in Dulbecco’s modified Eagle’s culture medium containing 10% fetal bovine serum (Life Technologies, Houston, TX, USA) and 1% penicillin and streptomycin (Life Technologies) at 37 °C in a humidified atmosphere of 5% CO_2_/95% air in a NuAire DH AutoFlow CO_2_ air-jacketed incubator (Plymouth, MN, USA).

Fertilized Ross 308 chicken eggs (*Gallus gallus domesticus*) from a local hatchery were placed in a humidified 37 °C incubator without CO_2_ to induce embryogenesis. After 7 days of egg incubation, a silicone ring containing 3 × 10^6^ MDA-MB-231 cells suspended in 20 μL of culture medium was placed on the chorioallantoic membrane (CAM) of the eggs. The eggs were incubated for an additional 7 days, then the tumors were resected for further analysis. The eggs were divided into 6 groups of 30 eggs each and were injected with 200 µL of test substances: 10 μg/mL M, 20 μg/mL GN and GO, and 2 complexes. The control group was treated with a saline solution. After 3 days, the tumors were resected for further analysis.

### 4.3. Measurement of Tumor Volume

Digital photos of tumors were taken using a stereo microscope (SZX10; Olympus Corporation, Tokyo, Japan) equipped with CellD software version 3.1. Measurements were completed using cellSens Dimension Desktop version 1.3 software (Olympus) and tumor volumes were calculated based on the measurements obtained.

### 4.4. Tumor Lysate

Tumors were placed in the lysis solution containing protease and phosphatase inhibitors (Sigma-Aldrich, St. Louis, MO, USA) according to the manufacturer’s instructions. Homogenization was performed using frozen metal balls and TissueLyser (QIAGEN, Hilden, Germany) at 50 Hz for 10 min on a shaking frozen cartridge. The samples were then centrifuged (30 min; 12,000 rpm; 4 °C), and the resulting supernatant was collected. We determined protein concentration using a bicinchoninic acid kit (Sigma-Aldrich, St. Louis, MO, USA).

### 4.5. Activity of Caspases

The activity of CASP3 and CASP8 was evaluated using ELISA tests according to the 96-plate microassay method with a p-Nitroaniline (pNA) calibration curve following the manufacturer’s protocol (n = 5 per group) (for CASP3–CASP3C; for CASP8–CASP8C, Sigma-Aldrich Company, St. Louis, MO, USA). Absorbance was measured at 405 nm using a microplate reader, and the activity of the tested caspases was calculated according to the protocol and presented as a percentage of activity compared to blanks marked as 0% activity.

### 4.6. Lipid Peroxidation Analysis

The influence of the tested complexes and their components on lipids was assessed using the Lipid Peroxidation (MDA) Assay Kit (MAK085; Sigma-Aldrich Company, USA). Samples (n = 5 per group), and appropriate reagents were handled according to the manufacturer’s protocol and incubated at 95 °C for 60 min. Then, the samples were chilled on ice for 10 min and transferred to a 96-well plate. Absorbance was measured at 532 nm using a microplate reader, and the concentration of MDA was calculated in nmol/mL.

### 4.7. SOD Activity

SOD activity was measured using the SOD Determination Kit (product number 9160-1kt-f). Samples (n = 5 per group) and appropriate reagents were handled according to the manufacturer’s protocol: both were transferred to a 96-well plate and incubated for 20 min at 37 °C. We measured absorbance at 450 nm using a Tecan Infinite 200 microplate reader (Tecan, Durham, NC, USA) and calculated the percentage of SOD activity.

### 4.8. GSH-Level Analysis

We assessed GSH levels in tumor cells by quantifying non-protein -SH groups in protein-depleted samples using Ellman’s method [87]. To deproteinate, 50% TCA (78.96 μL) was added to the supernatants (1.5 mL), and the samples were centrifuged at 3000 rpm for 5 min.

The deproteinated supernatants (25 μL) were then directly mixed with 0.2 M phosphate buffer pH 8.0 (200 μL) and 6 × 10^−3^ M DTNB (25 μL) in a 96-well plate (n = 5 per group). The absorbance was measured at 412 nm using a Tecan Infinite 200 microplate reader, and a standard curve was generated using different concentrations (0–75 nmol/mL) of GSH in 2.5% TCA.

### 4.9. Measurement of 8-Hydroxy-2′-Deoxyguanosine (8-OHdG) Concentration

The Deoxyguanosine (8-OHdG) ELISA Kit (abx257146, Abbexa Ltd., United Kingdom), is based on competitive enzyme-linked immuno-sorbent assay technology and is used to determine the level of DNA oxidation. Tumor lysates were diluted to equal the lowest concentration obtained after isolation, and five 50 µL samples were added to a 96-well plate. Fluids were then removed from the wells, and 50 µL of Detection Reagent A working solution was added. After incubation for 1 h at 37 °C, the wells were washed 3 times. Then, 100 μL of Detection Reagent B working solution was added to the wells and incubated for 30 min at 37 °C. Next, the wells were rinsed 5 times, and 90 μL of TMB Substrate was added and incubated for 15 min at 37 °C. Finally, 50 μL of Stop Solution was added, and the absorbance was measured at 450 nm using a Tecan Infinite 200 microplate reader (Tecan, Durham, NC, USA). The results were read according to the prepared standard curve as ng/mL.

### 4.10. Cytokine Protein Level

The level of cytokine proteins in tumors after exposure to the tested complexes and their individual components was examined using an antibody array (ab133998; Abcam, Cambridge, UK). We performed the assay in accordance with the manufacturer’s instructions using lysates containing 500 μg/mL of total protein per membrane. The membranes were visualized using the Azure Biosystem C400 (Azure, Dublin, CA, USA). The membrane photos were analyzed in ImageJ using the Protein Array Analyzer plugin. The results were normalized and compared to a dots control sample.

### 4.11. Statistical Analysis

Data were analyzed using multifactorial and monofactorial analysis of variance with Statgraphics^®^ 19 (StatPoint Technologies, Warrenton, VA, USA). The differences between groups were tested using Tukey’s multiple range tests. All mean values are presented with the standard deviation. Differences with *p* < 0.05 were considered significant.

## 5. Conclusions

The study demonstrated differences in the response to treatment with M, MGN, and MGO complexes. Analysis of the activity of CASP3 and CASP8 suggested that MGO may act on the external structures of the cell, while MGN mainly affects intracellular structures. The use of GN nanomaterials did not result in a major increase in oxidative stress markers, such as M alone did; however, MGN caused great oxidative damage to DNA. MGN also increased the levels of cytokines responsible for inhibiting breast cancer tumor progression and metastasis to a greater extent than the MGO complex. These findings suggest that there is a potential for using GN as a carrier for triple-negative breast cancer tumors.

## Data Availability

The data presented in this study are available on reasonable request from the first author.
